# Simulated scenarios in nursing: an integrative literature review

**DOI:** 10.1590/0034-7167-2022-0123

**Published:** 2022-11-28

**Authors:** Gustavo Correa de Amorim, Fabiana Cristina Pires Bernardinelli, Juliana da Silva Garcia Nascimento, Ingrid Fidelix de Souza, Divanice Contim, Suzel Regina Ribeiro Chavaglia

**Affiliations:** I Universidade Federal do Triângulo Mineiro. Uberaba, Minas Gerais, Brazil; II Universidade de São Paulo. Ribeirão Preto, São Paulo, Brazil

**Keywords:** Students, Nursing, Nurses, Simulation Training, Teaching, Learning, Estudiantes de Enfermería, Enfermeras y Enfermeros, Simulación, Enseñanza, Aprendizaje, Estudantes de Enfermagem, Enfermeiras e Enfermeiros, Simulação, Ensino, Aprendizagem

## Abstract

**Objectives:**

to identify in scientific literature which simulated clinical scenarios were developed and validated for teaching and learning in nursing.

**Methods:**

integrative review, carried out in seven sources of information. The Rayyan program was used for selection, content analysis to explore the findings and the methodological assessment tool of the validity process, entitled Quality Appraisal tool for Validity Studies.

**Results:**

initially, 1,179 manuscripts were identified and 14 were part of the sample. Two categories were defined: Profile of simulated clinical scenarios produced in nursing; and Clinical skills developed and their assessment mechanisms.

**Final Considerations:**

there was a preponderance of high-fidelity scenarios, built in Brazil in the last five years, aimed at nursing students on the themes of emergency, maternal care and stomatherapy, addressing the assessment of cognitive, psychomotor and affective skills in nursing. Most studies obtained good methodological quality in their content validity process.

## RESUMO

**Objetivos:**

identificar na literatura científica quais os cenários clínicos simulados desenvolvidos e validados para o ensino e aprendizagem em enfermagem.

**Métodos:**

revisão integrativa, realizada em sete fontes de informação. Utilizaram-se o programa Rayyan para seleção, a análise de conteúdo para exploração dos achados e a ferramenta de avaliação metodológica do percurso de validação, intitulada Avaliação da Qualidade para Estudos de Validade.

**Resultados:**

identificaram-se, inicialmente, 1.179 manuscritos e 14 compuseram a amostra. Definiram-se duas categorias: Perfil dos cenários clínicos simulados produzidos em enfermagem; e Habilidades clínicas desenvolvidas e seus mecanismos de avaliação.

**Considerações Finais:**

houve preponderância de cenários de alta fidelidade, construídos no Brasil, nos últimos cinco anos, voltados a estudantes de enfermagem sobre as temáticas da urgência e emergência, cuidado materno e estomaterapia, abordando a avaliação das habilidades cognitivas, psicomotoras e afetivas em enfermagem. A maioria dos estudos obteve boa qualidade metodológica em seu processo de validação de conteúdo.

## RESUMEN

**Objetivos:**

identificar en la literatura científica qué escenarios clínicos simulados fueron desarrollados y validados para la enseñanza y el aprendizaje en enfermería.

**Métodos:**

revisión integradora, realizada en siete fuentes de información. Se utilizó el programa Rayyan para la selección, el análisis de contenido para explorar los hallazgos y la herramienta de evaluación metodológica del proceso de validación, denominada *Quality Appraisal tool for Validity Studies*.

**Resultados:**

inicialmente se identificaron 1.179 manuscritos y 14 formaron parte de la muestra. Se definieron dos categorías: Perfil de escenarios clínicos simulados producidos en enfermería; y Habilidades clínicas desarrolladas y sus mecanismos de evaluación.

**Consideraciones Finales:**

hubo predominio de escenarios de alta fidelidad, construidos en Brasil en los últimos cinco años, dirigidos a estudiantes de enfermería sobre las temáticas de urgencia y emergencia, cuidado materno y estomaterapia, abordando la evaluación de las habilidades cognitivas, psicomotoras y afectivas en enfermería. La mayoría de los estudios obtuvieron buena calidad metodológica en su proceso de validación de contenido.

## INTRODUCTION

Active teaching and learning strategies, capable of encouraging the development of skills and attitudes, constitute a challenge for professors today^(1-2)^. Among the educational possibilities in nursing education, clinical simulation stands out, defined as a teaching strategy, guided by experiential learning, which replicates real situations, in a safe and controlled environment, to develop students’ cognitive (knowledge), psychomotor (procedural) and affective (attitudes/behavior) skills^(3-4)^.

Adopting the clinical simulation strategy requires the application of its steps, called preparation, participation and debriefing^(5)^. The preparation step is divided into pre-simulation phases, characterized by providing student with the necessary knowledge to experience the simulated scenario, associated with skills training and pre-briefing/briefing, an immediate phase to developing a scenario that covers environment clarification, learning objectives, clinical case and participant roles^(1,5)^.

The participation step covers developing a scenario simulated by students. Finally, debriefing configures an analytical process of discussion/reflection, usually carried out after the simulation scenario, in order to enhance the development of clinical skills^(1,6)^.

Experiencing a simulated scenario allows students to apply their knowledge, improve psychomotor skills in a controlled environment, make mistakes numerous times, without harming patients, and develop fundamental behavioral skills for work in nursing^(7)^. However, for this, it is necessary that the design of the adopted scenario is correctly aligned with the desired learning objectives and that it has been submitted to a validity process, in order to obtain clarity, realism and applicability towards teaching and learning^(8-10)^.

Despite the increasing clinical scenario use in nursing, it has not yet been possible to identify in the literature a study capable of synthesizing an overview of the scenarios already produced and validated. Moreover, no scientific evidence was found to describe which themes have been addressed through clinical simulation for the care of adult and older patients, the objectives and criteria that supported its construction. Thus, it is believed that this scientific gap may interfere in the determination of best practices in simulation-based teaching in nursing, given the lack of scientific evidence on what has already been advanced and what still needs to be done in the development of described and reliable clinical scenarios in this educational context^(1)^.

Moreover, knowledge synthesis on clinical scenario production and validity can encourage its use in nursing education, impact the quality of student and professional learning, patient safety and instigate the development of new scientific research capable of contributing to the progress of simulation-based teaching as an effective and innovative strategy^(11)^. Considering the above, the question is: what are the simulated clinical scenarios developed to promote the teaching and learning of nursing students and professionals?

## OBJECTIVES

To identify in the scientific literature which simulated clinical scenarios were developed and validated for teaching and learning in nursing.

## METHODS

This is an integrative literature review with the intention of synthesizing and critically assessing studies on simulated clinical scenarios, aimed at teaching and learning in nursing, supported by Preferred Reporting Items for Systematic Reviews and Meta-Analyses (PRISMA), a theoretical-methodological framework based on a four-step flowchart and a 27-item checklist capable of directing the correct performance of review studies^(12)^.

To develop the study, six steps were taken: (1) definition of theme and guiding question of research; (2) establishment of inclusion and exclusion criteria that will compose the sample; (3) categorization of information to be extracted from the studies; (4) assessment of included studies; (5) critical interpretation of results; and (6) synthesis of the data obtained^(13)^.

In the first step, the guiding question was formulated using the Patient-Intervention-Outcomes (PIO) strategy, a variation of the Patient-Intervention-Comparation-Outcomes (PICO) strategy^(14)^, considering as the acronym P (Population) nursing students and professionals; the acronym I (Intervention), the identification of simulated clinical scenarios aimed at nursing and the acronym O (Outcome), nursing education based on clinical simulation. Thus, the following question was elaborated: what are the simulated clinical scenarios developed and validated to promote the teaching and learning of nursing students and professionals?

In the second step, the criteria for inclusion and exclusion of articles were established, including primary methodological studies that addressed the development of simulated clinical scenarios for the care of adults and older adults, aimed at nursing students and professionals, without delimiting language or time frame, published in scientific journals, electronically. We excluded studies such as literature review, letter to the editor, editorials, case reports, abstracts published in annals of events, personal opinions, dissertations, theses, book chapters, institutional manuals and articles on virtual and hybrid simulated scenarios.

The following sources of information have been adopted: Medical Literature Analysis and Retrieval System Online (MEDLINE/PubMed^®^), Latin American and Caribbean Literature in Health Sciences (LILACS), Scopus, Cumulative Index to Nursing and Allied Health Literature (CINAHL), Web of Science, Excerpta Medica Database (EMBASE) and Education Resources Information Center (ERIC).

The search for evidence took place on November 23, 2021, based on the structural elements of the PIO strategy, to determine the descriptors and keywords. The descriptors obtained from the Descriptors in Health Sciences (DeCS) and Medical Subject Headings (MeSH) were used, in a trilingual way, their synonyms, in the plural and singular, and the Boolean operators. Knowing that each information source responds to different commands and works in a unique way. The search strategy was adapted, as exemplified below in Chart 1.

**Chart 1:** Search strategy, descriptors and keywords used in this integrative literature review, Uberaba, Minas Gerais, Brazil, 2022

Database	Descriptors	Search strategy
MEDLINE/PubMed^®^ Scopus	MeSH controlled descriptors: Nurses; “Students, Nursing”; “Simulation Training”, the keyword: Scenario and synonyms “Nurse; “Pupil Nurses”; “Student, Nursing”; “Nurses, Pupil”; “Nurse, Pupil”; “Pupil Nurse”; “Nursing Student”; “Nursing Students”; “Training, Simulation”	(“Nurses” OR “Nurse” AND “Students, Nursing” OR “Pupil Nurses” OR “Student, Nursing” OR “Nurses, Pupil” OR “Nurse, Pupil” OR “Pupil Nurse” OR “Nursing Student” OR “Nursing Students” AND “Simulation Training” OR “Training, Simulation” AND “Scenario” AND “Teaching” AND “Learning”).
LILACS	DeCS present controlled descriptors in Portuguese: “*Enfermagem”; “Estudantes de Enfermagem”; “Treinamento por Simulação”; “Ensino”; “Aprendizagem*” and the keyword scenario and its versions in English, Spanish and French.	**(“** *Enfermagem” AND “Estudantes de Enfermagem” AND “Treinamento por Simulação” AND “Cenário” AND “Ensino” AND “Aprendizagem*”). (“Nursing” AND “Students, Nursing” AND “Simulation Training” AND “Scenario” AND “Teaching” AND “Learning”). (“*Enfermería” AND “Estudiantes de Enfermería” AND “Entrenamiento Simulado” AND “Guión” AND “Enseñanza” AND “Aprendizaje”).* (“*Soins” AND* “*Élève infirmier” AND “Formation par simulation” AND “Scénario” AND “Enseignement” AND “Apprentissage”).*
CINAHL	Controlled descriptors in titles/subjects in English: Nurses; “Students, Nursing”; “Simulation Training”; Teaching; Learning. The keyword scenario was adopted.	(“Nurses” AND “Students, Nursing” AND “Simulations” AND “Scenario” AND “Teaching” AND “Learning”).
Web of Science	MeSH controlled descriptors in English: “Nurses”; “Students, Nursing”; “Simulation Training”; “Teaching”; “Learning” and the keyword scenario.	TS= (Nurses AND Students, Nursing^*^ AND Simulation Training^*^ AND Scenario AND Teaching AND Learning).
Embase	MeSH controlled descriptors in English: “Nurses”; “Students, Nursing”; “Simulation Training”; “Teaching”; “Learning” and the keyword scenario.	(((‘nurses’/exp OR nurses OR ‘nurse’/exp OR nurse) AND (‘students, nursing’/exp OR ‘students, nursing’) OR ‘pupil nurses’ OR ‘student, nursing’ OR ‘nurses, pupil’ OR ‘nurse, pupil’ OR ‘pupil nurse’ OR ‘nursing student’/exp OR ‘nursing student’ OR ‘nursing students’/exp OR ‘nursing students’) AND (‘simulation training’/exp OR ‘simulation training’) OR ‘training, simulation’) AND scenario AND (‘teaching’/exp OR teaching) AND (‘learning’/exp OR learning).
ERIC	Thesaurus controlled descriptors in English: “Nurses”; “Students, Nursing”; “Simulation Training”; “Teaching”; “Learning” and the keyword scenario.	(“Nurses” OR “Nurse” AND “Students, Nursing” OR “Pupil Nurses” OR “Student, Nursing” OR “Nurses, Pupil” OR “Nurse, Pupil” OR “Pupil Nurse” OR “Nursing Student” OR “Nursing Students” AND “Simulation Training” OR “Training, Simulation” AND “Scenario” AND “Teaching” AND “Learning”).

*MEDLINE/PubMed - Medical Literature Analysis and Retrieval System Online; LILACS - Latin American and Caribbean Literature in Health Sciences; CINAH - Cumulative Index to Nursing and Allied Health Literature; Embase - Excerpta Medica Database; ERIC - Education Resources Information Center.*

In the second step, the selection of studies was carried out, firstly by reading titles and abstracts, by two independent researchers, through a free, single-version web review program called Rayyan Qatar Computing Research Institute (Rayyan QCRI), due to its ability to facilitate the initial screening of manuscripts, exclude duplicate articles, and incorporate a high level of usability and selection effectiveness, with assistant researcher blinding^(15)^. Subsequently, the selected studies were read in full and their reference lists were checked for the inclusion of new articles, obtaining the desired final sample.

A data collection instrument previously validated^(16)^ was developed considering author, year of publication, country of origin, objective and main results. Moreover, quality assessment of studies’ methodological course was carried out through a specific tool for this purpose, entitled Quality Appraisal tool for Validity Studies (QAVALS), adopted internationally^(17)^ and nationally^(1)^, even if not yet validated for Brazilian Portuguese, as it is easy to interpret, handle, and reliable and does not generate an interpretation-dependent score^(17)^.

The QAVALS is composed of 24 criteria, which assess aspects of validity studies’ methodological quality, classified as “yes”, “no” or “other” (other= ND= not determined; NA= not applicable; NR= not reported). The more criteria that are met by the study and receive a “yes” rating, the better the validity quality will be^(17)^.

In the fourth step, the findings obtained were analyzed using thematic analysis, in three phases: (1) pre-analysis, with a thorough reading of evidence; (2) organization of convergent information and exploration of findings with clustering of convergences; and (3) data processing, listing the categories^(18)^. In the fifth and sixth steps, the information obtained was interpreted, presenting knowledge synthesis.

## RESULTS

**Figure 1:**
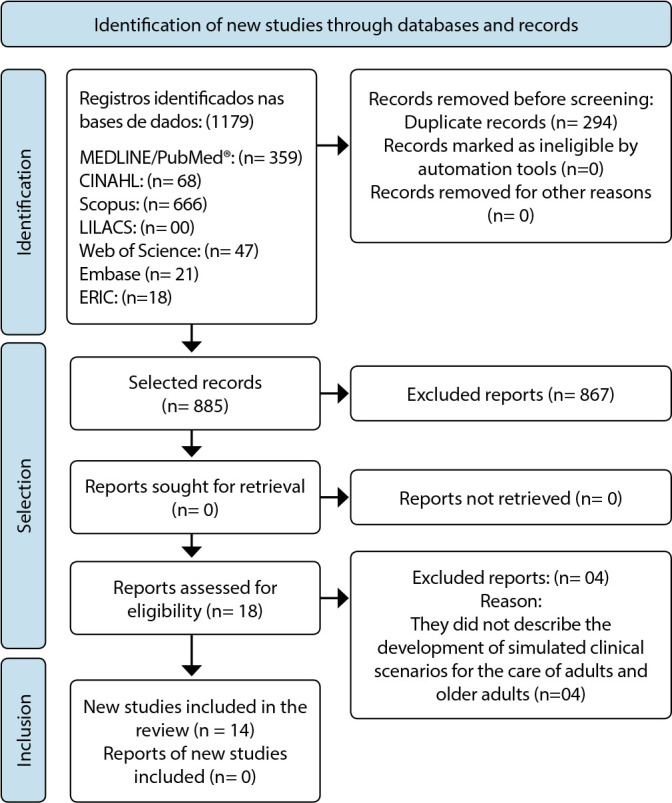
Flowchart of identification, selection and inclusion of studies, according to recommendations from the Preferred Reporting Items for Systematic Reviews and Meta-Analyses, Uberaba, Minas Gerais, Brazil, 2022

Initially, 1,179 primary studies were identified and 14 comprised the final sample. The selection process was demonstrated in Figure 1, as recommended by the PRISMA checklist^(12)^.

*MEDLINE/PubMed - Medical Literature Analysis and Retrieval System Online; LILACS - Latin American and Caribbean Literature in Health Sciences; CINAH - Cumulative Index to Nursing and Allied Health Literature; EMBASE - Excerpta Medica Database; ERIC - Education Resources Information Center.*

*Source: Page et al., 2020^(12)^.*

Then, the studies included in the sample were characterized according to their authorship, origin, year of publication, intention, main results, as shown in Chart 2 below.

**Chart 2:** Characterization of studies that composed the sample of this integrative literature review, Uberaba, Minas Gerais, Brazil, 2022

Author, year and origin	Objective	Main results
Gouveia et al., 2021^(19)^, Brazil.	Build and validate a scenario for the development of diagnostic reasoning skills in nursing students.	Eight judges validated the scenario content and obtained an agreement rate of 96%. Scenario components: prior knowledge of students; goals; theoretical foundation; theme; date; responsible; scenario complexity; expected interventions; expected results; faithfulness; clinical cases for facilitator to student and actor; vital parameters; reason for hospitalization; medical prescription; materials; characterization of actors; physical space; human resources; scenario time; validity; development; debriefing; and assessment.
Carreiro; Romão; Costa, 2021^(20)^, Brazil.	Build and validate two medium-fidelity clinical simulation scenarios in basic life support in the context of primary care.	A scenario of cardiorespiratory arrest in primary care and airway obstruction by a foreign body was validated in content, by seven judges, obtaining a CVI between 85.7% and 100%. Scenario components: participant’s previous experience; goals; scenario duration; human Resources; theme; faithfulness; clinical case; physical exam; conduct; actor characterization; materials; physical space; development; debriefing; and assessment.
Santana et al., 2021^(21)^, Brazil.	Build and validate the content of a clinical simulation scenario for teaching in-hospital transport of critically ill patients.	The Delphi technique was adopted to assess inter-rater agreement, obtaining 80% agreement through five judges. Scenario components: theme; target Audience; prerequisites; number of students; scenario; time; goals; pre-briefing/briefing; clinical case; high-fidelity scenario and mannequin preparation; materials; necessary actions; debriefing; and references.
Rocha et al., 2021^(22)^, Brazil.	Validate simulated scenarios for teaching and learning nursing students about pressure injury assessment and treatment.	Two scenarios were validated for content by ten judges: the first on nursing care in the assessment of pressure injuries to hospitalized patients; and the second about nursing care in the treatment of pressure injuries to the bedridden patient at home, resulting in an overall Scale-Level Content Validity Index greater than 0.80. Scenario components: previous knowledge of students; goals; theoretical foundation; responsible; scenario fidelity; documentation; clinical case; material and human; team training; debriefing; and assessment.
Almeida et al., 2021^(23)^, Brazil	Validate scenarios for application in stomatherapy care.	The study was validated by the CVI of 96% agreement among five judges. The scenarios were structured with general and specific objectives, fidelity, problem solving, clues, assessment checklist, pre-briefing and briefing.
Fonseca et al., 2020^(24)^, Brazil.	Validate a maternal-infant simulation scenario on humanized childbirth and birth.	A level of agreement above 80% was obtained in all aspects assessed by 31 experts. Scenario components: learning objectives; necessary resources; pre-briefing and debriefing guidelines; simulated situation, participant and role description; and checklist of expected actions.
Carvalho; Zem-Mascarenhas, 2020^(25)^, Brazil.	Build, validate and test a high-fidelity clinical simulation scenario for the management of sepsis.	The scenario obtained an CVI greater than 0.90 by nine judges. Scenario components: title; public; prior knowledge; simulation modality; simulation site; materials; simulator types; simulation proposal; introduction; time, design; simulation experience; pre-briefing; debriefing; participants; and expected results.
Souza et al., 2020^(26)^, Brazil.	Validate a scenario for the prevention of bloodstream infections associated with peripheral venous catheters.	The study showed that all the simulated clinical scenario requirements reached agreement among the 12 judges above 80% regarding clarity and relevance. Scenario components: objectives; team and participants; materials, equipment and props; faithfulness; assessment method; pre-briefing; and debriefing.
Andrade et al., 2019^(27)^, Brazil.	Build and validate a clinical simulation scenario for postpartum hemorrhage.	The items assessed by the 22 judges had a CVI > 0.90, and in the assessment by students, CVI > 0.95. Scenario components: learning objectives; faithfulness; assessment instrument; activities developed before the scenario; and debriefing.
Negri et al., 2019^(9)^, Brazil.	Build and validate a scenario on nursing care for colostomy patients.	Nine experts obtained an agreement rate of 100%. Scenario components: previous experience; goals, time; prior reading material; human Resources; scenario preparation; scenario planning, materials and documentation; development; debriefing; and assessment.
Gonçalvez-Meska et al., 2019^(28)^, Brazil.	Build and validate four simulated clinical scenarios in care involving the presence of unpleasant odors.	Five judges and 15 undergraduate nursing students validated the scenario in terms of content, obtaining 100% agreement. Scenario components: behavioral guidance, resource recognition, pre-briefing; and debriefing. Four scenarios were validated: care of a patient who presents vomiting; another with diaper evacuation; one with an infected pressure ulcer; and one patient with colostomy.
Leon et al., 2018^(29)^, Brazil.	Describe the construction of two clinical cases and validate them for use in the realistic maternal-infant simulation.	The scenarios developed were about safe patient care, validated by five judges, with patient decision-making and self-care preparation validated by six judges. Both obtained CVI > 0.80. Scenario components: objectives; duration; participants; simulator, materials, prerequisites for participation; and clinical case.
Eduardo et al., 2016^(30)^, Brazil.	Validate a scenario on waste management from health services.	Three judges validated the scenario, which obtained 100% inter-rater agreement. Scenario components: responsible for the scenario; target audience; learning objectives; expected actions; duration; place; participants; simulator; patient characteristics; equipment; materials; prerequisites to participate; and clinical case.
Jung et al., 2015^(31)^, South Korea.	Develop and validate a scenario to improve patient safety during asthma care.	A total of ten judges validated this clinical scenario, which obtained a Content Validity Coefficient > 0.80. Scenario components: determination of objectives; content development; preparation; application; and assessment.

*CVI - Content Validity Index.*

Most of manuscripts included in the sample of this research were from the last five years^(9,19-29)^, validated in content by more than eight experts^(9,19,22,24-27,31)^, with a level of inter-rater agreement above 80%^(9,19-31)^. Two studies were submitted to the Delphi technique^(21-22)^, and only one article was international^(31)^.

The main components that structured the design of simulated clinical scenarios were: (1) learning objectives^(9,19-20,22-24,26-27,30-31)^; (2) scenario fidelity level^(9,19-20,22-23,26-27)^; (3) clinical case^(19-20,22,24,30)^; (4) materials used^(9,19-22,25-26,29-30)^; and scenario duration^(9,19-21,25,29-30)^. It is also worth considering that there was a preponderance of studies^(9,19-28)^ that considered the other simulation steps, such as preparation and debriefing, as components of the simulated scenario design.

The findings allowed the elaboration of two categories: *Profile of simulated clinical scenarios produced in nursing; Developed clinical skills and their assessment mechanisms.*

Category 1 deals with the presentation of the profile of the simulated scenarios that have been produced for nursing education, covering:

- Learning themes: on emergency: basic life support in the context of primary care^(20)^; intra-hospital transport of critical patients^(21)^; asthma care^(31)^; about maternal care: childbirth and humanized birth^(24)^; postpartum hemorrhage^(27)^; licit and illicit drug use and early sexual initiation, pregnancy and abortion^(29)^; on the care of wounds and stomas: assessment and treatment of pressure injuries in nursing^(22)^; patient care with stomatherapy^(23)^; nursing care for colostomy patients^(9)^; on infection: management of sepsis^(25)^; prevention of infections associated with peripheral catheters^(26)^; on different topics: nursing diagnosis reasoning^(19)^; management of waste from health services^(30)^; care for patients with the presence of unpleasant odors^(28)^.
- Target audience: nursing students^(9,19-20,22,28-31)^; professional nurses and nursing students^(21,25,27)^; professional nurses^(23-24,26)^;
- Theoretical frameworks that supported the simulation: National League Nursing Jeffries Simulation Theory (NLN/JST)^(23-26)^; International Nursing Association for Clinical Simulation and Learning (INACSL)^(22,24-26)^; theoretical-practical script for clinical simulation proposed by Fabri^(9,22,28)^; Bloom’s Taxonomy^(9,20,28)^;
- Scenario fidelity: high fidelity^(9,19,21-25,29-31)^; medium fidelity^(20,27-28,30)^; low fidelity^(26)^;
- Instrument adopted: mannequin^(21,24,26,30-31)^; simulated patient^(9,22,24-25,28-29)^; standardized patient^(19-20,23-25)^;
- Scenario duration: 10 minutes^(21,24-25,29-30)^; 15 minutes^(19,23,26,28)^; 20 minutes^(9,27,31)^; 30 minutes^(20)^.

Category 2 addressed the type of clinical skill that the simulated scenario proposed to develop and its assessment mechanisms:

- Cognitive skills/knowledge^(9,19-31)^;
- Psychomotor/procedural skills^(9,19-31)^;
- Affective/attitudinal skills: decision-making^(21,23-24,26,28-29,31)^; self-confidence^(9,20-22,25-26)^; clinical judgment^(21,24-25,31)^; satisfaction^(9,20,22,26)^; critical thinking^(19,24-26)^; and reflection^(25-27)^.

To assess knowledge, theoretical assessment with objective questions^(9,26)^ and the Pieper knowledge test were adopted^(22)^. For psychomotor assessment, the Objective Structured Clinical Examination (OSCE) was used^(23)^. To assess the attitudinal aspects, the Student Satisfaction and Self-Confidence in Learning Scale^(9,20,22)^, the Satisfaction with Simulated Clinical Experiences Scale^(9,22)^, the Self-Confidence Assessment Scale for Emergency Action^(20)^, the Diagnostic Reasoning Inventory^(19)^ and the Lasater Clinical Judgment Rubric - Brazilian Version^(25)^.

As these are methodological studies, it was considered important to present quality assessment of the validity process performed by studies included in the sample of this research, adopting the QAVALS tool^(17)^, as noted in Chart 3.

**Chart 3:** Methodological quality assessment of sample validity studies using the Quality Appraisal tool for Validity Studies, Uberaba, Minas Gerais, Brazil, 2022

Items	Studies													
	9	19	20	21	22	23	24	25	26	27	28	29	30	31
1. Was the study design reported?	Y	Y	Y	Y	Y	Y	Y	Y	Y	Y	Y	Y	Y	Y
2. Did the study provide an accurate description of the type of validity tested?	Y	Y	Y	Y	Y	Y	Y	Y	NR	Y	Y	Y	Y	Y
3. Was the study setting and time frame of participant recruitment clearly outlined and described?	Y	Y	Y	Y	Y	Y	Y	Y	N	Y	Y	Y	Y	Y
4. Were the criteria for participant selection clearly described?	Y	Y	Y	Y	Y	Y	Y	Y	N	Y	Y	Y	Y	Y
5. Were the participants in the study representative of the sample population from which they were recruited?	Y	Y	Y	Y	Y	Y	Y	Y	NR	Y	Y	Y	Y	Y
6. Did the study clearly describe the outcome measures to be validated?	Y	Y	Y	Y	Y	Y	Y	Y	N	Y	Y	Y	Y	Y
7. Did the study provide a clear description of the procedures for testing validity?	Y	Y	Y	Y	Y	Y	Y	Y	Y	Y	Y	Y	Y	N
8. Was the testing procedure standardized for all participants?	Y	Y	Y	Y	Y	Y	Y	Y	Y	Y	Y	Y	Y	NR
9. Was a priori sample size calculation performed to ensure that the study had sufficient power?	N	N	N	N	N	N	N	N	N	Y	N	N	N	N
10. Did the study describe and justify any attrition that may have occurred?	NR	NR	NR	NR	NR	NR	NR	NR	NR	NR	NR	NR	NR	NR
11. Were the statistical analyses used to test validity appropriate for the study?	Y	Y	Y	Y	Y	Y	Y	Y	Y	Y	Y	Y	Y	NR
12. When multiple comparisons were performed, were appropriate statistical adjustments used to control for the likelihood of a type 1 error?	NA	NA	NA	NA	NA	NA	NA	NA	NA	NA	NA	NA	NA	NA
13. Did the study identify potential confounding variables and if so, were measures taken to adjust for these confounders?	NA	NA	NA	NA	NA	NA	NA	NA	NA	NA	NA	NA	NA	NA
14. Were the primary findings of the study clearly described?	Y	Y	Y	Y	Y	Y	Y	N	Y	Y	Y	N	Y	N
15. Were validity coefficients reported for primary outcomes?	Y	N	N	N	Y	Y	Y	Y	N	Y	Y	Y	Y	N
16. For primary outcomes, did the study report the standard deviation or confidence intervals for normally distributed data? Or, if non-normally distributed data, did the study report the inter-quartile range for the main outcomes?	N	N	N	N	N	N	N	N	Y	Y	N	N	N	N
17. Was the process of selecting expert panel and their qualifications described?	Y	Y	Y	Y	Y	Y	Y	Y	Y	Y	Y	Y	Y	Y
18. Did the study provide a rationale for the selection of the reference standard?	NA	NA	NA	NA	NA	NA	NA	NA	NA	NA	NA	NA	NA	NA
19. When the index test was assessed by more than one rater, were the raters blinded to the findings of the other raters?	NA	NA	NA	NA	NA	NA	NA	NA	NA	NA	NA	NA	NA	NA
20. When the index test was assessed by more than one rater, was the inter-rater reliability between raters established and reported?	NA	NA	NA	NA	NA	NA	NA	NA	NA	NA	NA	NA	NA	NA
21. Was the time interval used between administration of reference standard and the test measure appropriate?	NA	NA	NA	NA	NA	NA	NA	NA	NA	NA	NA	NA	NA	NA
22. Were subjects in different groups homogenous at baseline or if they weren’t homogenous at baseline, were differences between groups accounted for during the analysis?	NA	NA	NA	NA	NA	NA	NA	NA	NA	NA	NA	NA	NA	NA
23. Did the measures used for convergent validity represent a similar construct as the outcome measure of interest?	NA	NA	NA	NA	NA	NA	NA	NA	NA	NA	NA	NA	NA	NA
24. Did the measures used for discriminant validity represent a construct different from the outcome measure of interest?	NA	NA	NA	NA	NA	NA	NA	NA	NA	NA	NA	NA	NA	NA

*NA - not applicable; NR - not reported; Y - yes; N - no.*

Most of the studies that made up the sample included most of the validity criteria relevant to content assessment, demonstrating good methodological quality^(9,19-25,27-30)^. Only two articles did not meet a diversity of criteria^(26,31)^. It should be noted that, because scenario validity is content, the classification “not applicable” (NA), indicated for criteria such as criterion validity, construction validity for known groups, convergent construction validity and construction validity discriminant, did not interfere in assessing studies’ methodological quality.

Given the above, the validity criteria of greater fragility were the calculation of the sample size of participants to carry out the pilot test of the scenarios with the target audience, the description of friction during validity and result description of validity coefficient and standard deviations or confidence intervals.

## DISCUSSION

Intensive clinical simulation use by nursing in contemporary times has increasingly demanded clinical scenario design construction and validity, capable of optimizing the development of desired professional clinical skills and providing greater realism, bringing students closer to the contexts experienced in real situations^(32)^.

This study gives the science of nursing an originality, as it presents an overview of clinical scenario use, capable of supporting teaching and learning based on simulation, demonstrating the themes, contexts, learning intentions and assessment mechanisms that have already been considered in this scope, to highlight the advances and also the gaps that can be explored. Also, for critically assessing the manuscripts included, in their validity process and pointing out the existing methodological strengths and weaknesses, aiming at the future elaboration of more robust studies on clinical scenarios in nursing.

It is important to highlight the relevance of all studies identified on this topic and the preponderance of national literature on clinical scenario validity^(9,19-30)^, given that, in Brazil, there is a tendency to practice clinical scenario construction and validity aimed at simulated teaching in nursing and its presentation in scientific articles^(20-22,25)^, differing from simulation research in the international context, which performs scenario validity by experts during the methodological path, but generally does not consider its detailed description in the studies^(33-35)^.

Another relevant finding of this review is that most studies^(9,19-28)^ identified inserts all steps of clinical simulation (preparation, participation and debriefing) as simulated scenario elements. Thus, there is a scarcity in literature on the elaboration and validity of more complete simulation designs, with the presentation of simulation steps separately, with the intention of guiding, facilitators and professors, in a clear and didactic way, regarding the planning and application of simulated activities in nursing^(1,5,32)^.

It was possible to understand the profile of the simulated clinical scenarios already developed for the care of adult and older patients in nursing, based mainly on emergency^(20-21,31)^, maternal care^(24,27,29)^ and wound and stoma care contexts^(9,22-23)^. In the meantime, the findings identified in this review made it evident that, although simulated scenario use is already considered a successful practice for teaching in nursing^(9,19-28)^, which can enhance, exponentially, learning in nursing^(36)^, there is still a need to extend its application to the teaching of other topics, which may take advantage of the benefits of this pedagogical strategy in the development of clinical skills^(1)^.

Most clinical scenarios^(23-26)^ discussed here based their construction on consistent theoretical-methodological frameworks, especially the National League Nursing Jeffries Simulation Theory (NLN/ JST). A study carried out in Brazil, which aimed to build and validate three clinical scenarios and report the application with candidates for the title of expert in stomatherapy, adopted the Jeffries Simulation Theory, contemplating the elements determined by this conceptual model of simulation: facilitator, student, educational practices, simulation design and expected results. It was observed that the chosen design allowed the candidates for the title of expert to demonstrate their knowledge in the area and achieve the desired objectives^(23)^.

Many changes have occurred in simulation-based teaching following the release of the Jeffries Simulation Theory in 2005, due to the provision of a framework for this educational modality. In 2016, a new version of this theory was published with the intention of obtaining, after a deep literary search, more consistent and standardized simulation practices, capable of disseminating knowledge and conducting the planning of more effective simulated scenarios^(37)^.

A balance was observed in the adoption of simulators/mannequins, simulated patients (trained actors) and standardized (community members who take over the role of patient),to enable simulated teaching and the preponderance of a high level of fidelity in this context, related to the degree of realism achieved by the proposed simulated scenario design^(38)^.

A survey carried out in a regional school of nursing in South Korea corroborates this scenario, with the objective of improving decision-making, problem-solving and student communication about the care of asthmatic patients in the Emergency Care Unit, through the execution of a high-fidelity scenario, characterized by the articulation of an urgent and emergency environment close to the real one, which generated emotion in students, equipped with diverse materials and high-realism simulator^(31)^.

It is worth demystifying that one should not only value the simulator fidelity level to classify the degree of realism of a scenario, but a set of all dimensions, such as environmental (equipment, tools, simulators, makeup, noise, adornments), psychological (emotions, beliefs and self-awareness of participants) and social (motivation and goals of participants and instructors, group culture, degree of openness and trust as well as the way participants think) factors^(38)^.

In addition to the criteria already presented, we approached the execution time of clinical scenarios identified^(21,24-25,29-30)^, characterized by a duration of ten minutes by most studies, an execution time also adopted in a research carried out in a public nursing school, in the countryside of the state of São Paulo, to build and validate a clinical simulation scenario of high fidelity on nursing care to colostomy patients. The 10-minute simulated experience in this context was questioned by the judges during scenario validity, suggesting that the experience be ended only when it contemplated the proposed learning objectives^(9)^.

Thus, it is considered that, in the design planning of simulated clinical scenarios in nursing, learning objectives should be established first and, after content validity by experts, testing with the target audience, if possible, to determine accurately the time that will be programmed for the experience^(11)^.

Although most studies on simulated scenarios have proposed to develop participants’ cognitive, psychomotor and affective skills^(9,19-31)^, only one article^(23)^ identified a tool for assessing psychomotor skills in nursing, while other manuscripts did not report assessment instruments^(21,24,27-31)^. This is a methodological gap that can be filled by clinical scenario design elaboration and validity, capable of contemplating the participant assessment phase, in a global way, describing the way in which knowledge, practical skills, and attitudes and emotions of students will be assessed^(11)^.

Most studies covered showed good quality in the validity path adopted, which indicates greater reliability to replicate the clinical scenarios in nursing, produced until then, to support the simulated teaching^(9,19-25,27-30)^.

A review corroborates with this research, which intended to assess the validity process quality carried out in studies that developed simulated clinical scenarios for teaching and learning in nursing, through the QAVALS^(17)^, presenting six primary studies of good methodological quality, indicated by this tool^(1)^.

The clinical scenario validity process is essential for the practice of simulation in health, as it provides subsidies for the elements of a tool to become relevant and representative for fulfilling its purpose^(39)^. In the context of building clinical scenarios, content validity provides its scientific recognition, reproducibility and coherence, to achieve higher quality simulation-based teaching and learning in nursing^(27)^.

### Study limitations

A limitation of this review refers that the search was limited to primary published studies, i.e., the gray literature was not included. Other primary studies could be identified through searches in other databases and clinical trial registry websites as well as the inclusion of studies published in journals from different areas of health. In addition to this, using descriptor “nursing student” limited our search, making it impossible to identify another study on the investigated topic. The search for simulation use as a teaching and learning strategy in the continuing education of nursing professionals could have resulted in a greater number of clinical scenarios developed and validated.

### Contributions to nursing

This study contributes to the advancement of science in nursing, as it presents a contemporary profile of the construction and validity of clinical scenarios for this context and substantiates the choice of professors and facilitators about the best pedagogical practices in simulation. It is recommended the elaboration of new review studies, capable of investigating the production of clinical scenarios for all care areas as well as clinical trials to test the effectiveness of existing simulated scenarios.

## FINAL CONSIDERATIONS

Most simulated clinical scenarios in nursing, aimed at the care of adults and older adults, were produced and validated in the last five years, in Brazil, on the teaching of urgency and emergency, maternal care and stomatherapy, aimed at nursing students. Regarding the theoretical frameworks that supported scenario construction, Jeffries’ theoretical framework was highlighted, having learning objectives, fidelity level, clinical case, material resources and duration as main components. Although the simulated clinical scenarios are capable of developing and assessing cognitive, psychomotor and affective skills, it is necessary to accurately establish mechanisms and instruments used to analyze scenario construction and validity. Most of the manuscripts that made up the sample included the criteria of the validity process addressed by the QAVALS tool, demonstrating good methodological quality in scenario development.
